# Extension of Cellular Lifespan by Methionine Restriction Involves Alterations in Central Carbon Metabolism and Is Mitophagy-Dependent

**DOI:** 10.3389/fcell.2019.00301

**Published:** 2019-11-28

**Authors:** Jason D. Plummer, Jay E. Johnson

**Affiliations:** Department of Biology, Orentreich Foundation for the Advancement of Science, Cold Spring, NY, United States

**Keywords:** aging, ageing, longevity, healthspan, mitochondria, autophagy, methioninase, yeast

## Abstract

Methionine restriction (MR) is one of only a few dietary manipulations known to robustly extend healthspan in mammals. For example, rodents fed a methionine-restricted diet are up to 45% longer-lived than control-fed animals. Tantalizingly, ongoing studies suggest that humans could enjoy similar benefits from this intervention. While the benefits of MR are likely due, at least in part, to improved cellular stress tolerance, it remains to be determined exactly how MR extends organismal healthspan. In previous work, we made use of the yeast chronological lifespan (CLS) assay to model the extension of cellular lifespan conferred by MR and explore the genetic requirements for this extension. In these studies, we demonstrated that both dietary MR (D-MR) and genetic MR (G-MR) (i.e., impairment of the cell’s methionine biosynthetic machinery) significantly extend the CLS of yeast. This extension was found to require the mitochondria-to-nucleus retrograde (RTG) stress signaling pathway, and was associated with a multitude of gene expression changes, a significant proportion of which was also dependent on RTG signaling. Here, we show work aimed at understanding how a subset of the observed expression changes are causally related to MR-dependent CLS extension. Specifically, we find that multiple autophagy-related genes are upregulated by MR, likely resulting in an increased autophagic capacity. Consistent with activated autophagy being important for the benefits of MR, we also find that loss of any of several core autophagy factors abrogates the extended CLS observed for methionine-restricted cells. In addition, epistasis analyses provide further evidence that autophagy activation underlies the benefits of MR to yeast. Strikingly, of the many types of selective autophagy known, our data clearly demonstrate that MR-mediated CLS extension requires only the autophagic recycling of mitochondria (i.e., mitophagy). Indeed, we find that functional mitochondria are required for the full benefit of MR to CLS. Finally, we observe substantial alterations in carbon metabolism for cells undergoing MR, and provide evidence that such changes are directly responsible for the extended lifespan of methionine-restricted yeast. In total, our data indicate that MR produces changes in carbon metabolism that, together with the oxidative metabolism of mitochondria, result in extended cellular lifespan.

## Introduction

A methionine-restricted diet dramatically extends the healthspan of a variety of model organisms. Methionine-restricted rodents are up to 45% longer-lived than control-fed littermates, and similar improvements in longevity have been observed for flies and worms subjected to this intervention (Orentreich et al., 1993; Richie et al., 1994; Miller et al., 2005; Troen et al., 2007; Cabreiro et al., 2013; Lee et al., 2014). To gain insight into the molecular mechanisms underlying the healthspan benefits of methionine restriction (MR), we previously made use of multiple mammalian cell culture- and budding yeast-based model systems (Johnson and Johnson, 2014). Using the latter, we assessed the effect of MR on the ability of yeast to tolerate various cytotoxic stresses, as well as the effect of MR on chronological lifespan (CLS), defined as the period of time that yeast can remain viable in a non-dividing state (Fabrizio and Longo, 2007). Under certain conditions, the CLS assay is considered to model the aging of quiescent cell populations in higher organisms, but the production of certain toxic metabolites by stationary phase yeast means that CLS is often more accurately thought to be a measure of cellular stress resistance (Burtner et al., 2009). That said, using the above approaches, we found that MR confers stress tolerance to yeast and dramatically extends CLS (Johnson and Johnson, 2014). Moreover, these benefits involve mitochondrial retrograde (RTG) signaling, as deletion of the gene encoding the essential RTG stress signaling factor Rtg3 abrogates both the stress tolerance and extended longevity of methionine-restricted cells. In the same study, we also obtained evidence that MR engages similar stress responsive pathways in cultured mouse and human cells, improving their survival when subjected to multiple cytotoxic stresses, and delaying the onset of replicative senescence. Collectively, these results raised the possibility that mitochondrial signaling might underlie, at least in part, the benefits of MR to mammals.

While these studies suggested a putative role for mitochondria in supporting the longevity-promoting benefits of MR, many of the molecular mechanisms underlying these benefits remained unknown. Concurrent with our study, a number of other groups published work describing the extension of CLS by MR (Wu et al., 2013; Lee et al., 2014; Ruckenstuhl et al., 2014). Notably, Ruckenstuhl et al. (2014) described the formation of acidified vacuoles in yeast undergoing MR, and demonstrated a requirement of MR-induced CLS extension on core autophagy factors (Atg5, Atg7, and Atg8) and the vacuolar ATPase. A more recent study described the use of ribosome profiling and RNA-seq to assess translational and transcriptional changes in methionine-restricted yeast (Zou et al., 2017). Consistent with the results from Ruckenstuhl et al. (2014), the authors found that, among others, pathways involved in the induction of autophagy were differentially regulated in methionine-restricted cells. Indeed, this result nicely confirms the gene expression analyses from our previous report, which identified several factors involved in autophagy as being upregulated by MR (Johnson and Johnson, 2014). Together, these studies were strongly suggestive that a key component of the beneficial effects of MR on cell survival and longevity was the activation of autophagy.

In the current study, we show work aimed at elucidating the mechanistic relationship between autophagy and MR-dependent CLS extension. Consistent with previous studies, as well as the supposition that increased autophagic capacity is important for the benefits of MR, we show that loss of any of several factors involved in the initiation of autophagy abrogates the extension of CLS by MR. We also present epistasis analyses that provide further evidence that increasing autophagic capacity directly underlies the benefits of MR to yeast. Importantly, our data also clearly demonstrate that, of the many known types of selective autophagy, MR-mediated CLS extension requires only mitophagy, the autophagic recycling of mitochondria. As expected, given both this finding and our previous observation that the benefits of MR require an intact mitochondrial RTG signaling system, we demonstrate that functional mitochondria are required for the full benefit of MR to CLS. Finally, we observe that methionine-restricted yeast show substantial alterations in carbon metabolism, and provide evidence that such alterations contribute significantly to the extended lifespan of methionine-restricted yeast. Taken together, our data are consistent with MR producing changes in carbon metabolism that work in combination with the oxidative metabolism of mitochondria to directly improve cellular survival.

## Results

### Extension of CLS by MR Involves the Autophagy-Related Phosphatidylinositol-3-Kinase Complex I

**FIGURE 1:**
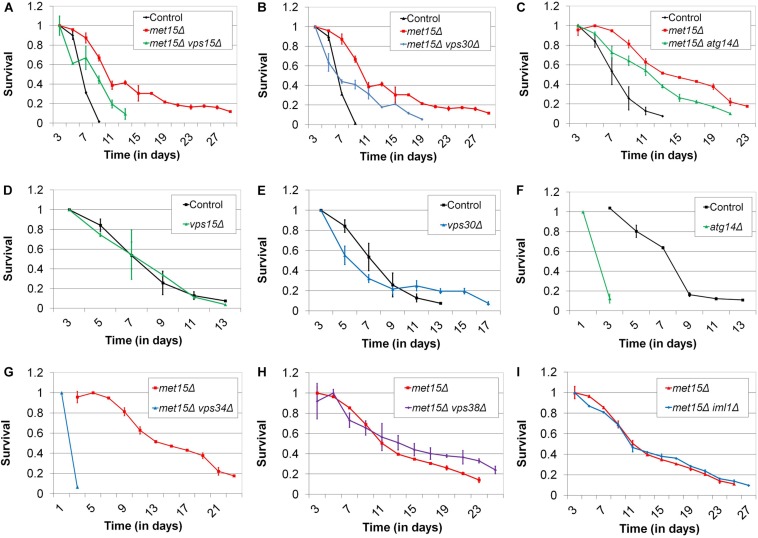
The autophagy-related PI3K complex I mediates MR-dependent CLS extension. Deletion of each of the genes encoding the autophagy-related PI3K complex I members **(A)** Vps15, **(B)** Vps30, and **(C)** Atg14 moderately reduces the CLS of methionine-restricted yeast. Conversely, otherwise wild-type cells deleted for the genes encoding **(D)** Vps15 and **(E)** Vps30 do not negatively impact CLS. **(F)** Otherwise wild-type cells lacking Atg14 and **(G)** methionine-restricted cells lacking Vps34 are extremely short-lived. Neither **(H)** Vps38 nor **(I)** Iml1 are required for maximal extension of lifespan by G-MR. For all panels, bars denote standard error of the mean (SEM).

In previous work, aimed at exploring the molecular mechanisms underlying the benefits of MR to yeast CLS and stress tolerance (Johnson and Johnson, 2014), we performed expression profiling of methionine-restricted yeast. These analyses revealed that a number of factors with roles in a wide range of biological processes are differentially expressed in methionine-restricted cells compared with controls. Of particular interest to us was the upregulation of several MR-responsive transcripts that encode factors involved in autophagy (*VPS15*, *VPS30*, *VPS34*, *ATG7*, *ATG9*, *ATG14*, *ATG17*, *ATG27*, *ATG29*, *ATG31*, *ATG33*, and *IML1*) (Johnson and Johnson, 2014). Indeed, a recent study from Zou et al. (2017) also found the pathways governing autophagy to be differentially regulated in methionine-restricted yeast. In addition, it has been demonstrated that the core autophagy factors Atg5, Atg7, and Atg8 are each indispensable for the MR-dependent extension of yeast CLS (Ruckenstuhl et al., 2014). A growing body of work has implicated autophagy in nearly all of the interventions that promote longevity in mammals, including calorie restriction, rapamycin treatment, sirtuin modulation, spermidine administration, and others (Eisenberg et al., 2009; Mercken et al., 2014; Madeo et al., 2015). For autophagy to have a phylogenetically conserved role in the regulation of longevity is perhaps not surprising given that the overwhelming majority of the interventions known to extend mammalian healthspan were first identified as promoting yeast survival. Prompted by such findings, we considered that the observed upregulation of some (or all) of the MR-responsive autophagy factors that we identified above might be involved in either activating autophagy or increasing autophagic capacity, in turn contributing to the extension of CLS observed for methionine-restricted cells. To explore this possibility, we assessed the lifespans of methionine-restricted cells deleted for many of the above autophagy factors, to determine which, if any, might abrogate the extended longevity associated with MR. For these experiments, the methionine-restricted state was produced *via* “genetic MR” (G-MR), which results from any of a handful of genetic manipulations (e.g., *met15Δ*) that compromise the cell’s methionine biosynthetic machinery, and which we previously demonstrated to confer both stress tolerance and extended cellular lifespan to yeast and cultured mammalian fibroblasts (Johnson and Johnson, 2014). We found that single deletions of *VPS15*, *VPS30*, and *VPS34* all significantly compromise the extended CLS typically associated with MR (*p* < 0.0001) (Figures 1A,B,G). Remarkably, in the case of cells lacking *VPS34*, MR fails to confer any benefit whatsoever to this mutant, which displays a slow growth phenotype and is one of the shortest-lived strains we have yet encountered; indeed, these cells fail to survive beyond 3 days in culture, the time-point at which CLS assays typically begin (Figure 1G). For cells lacking *VPS15* and *VPS30*, the loss of these factors does not result in a reduced CLS in an otherwise wild-type setting, demonstrating that the reduction in lifespan observed for *vps15Δ* and *vps30Δ* mutants under methionine-restricted conditions is due to impairment of MR-related benefits and not to non-specific sickness (Figures 1D,E). The median survival of *met15Δ atg14Δ* cells is significantly less than that of methionine-restricted control cells (*p* = 0.0107), although the reduction in their maximal lifespan only approaches significance (*p* = 0.0878) (Figure 1C). In addition, single mutant cells lacking only Atg14 are extremely short-lived (Figure 1F), raising the possibility that CLS impairment in *atg14Δ* cells might be a function of negative effects on both autophagy and important autophagy-independent functions of Atg14. With respect to the functions of Vps15, Vps30, Vps34, and Atg14, these factors are all members of the tetrameric phosphatidylinositol-3-kinase (PI3K) complex I, which localizes to the pre-autophagosome and vacuole, and is required for the initiation of essentially all autophagic processes (Kihara et al., 2001; Wen and Klionsky, 2017). Vps34 is the catalytic subunit that possesses PI3-kinase activity, whereas the three other factors regulate its activity in various ways. Thus, it might be expected that Vps34 would be the most critical member of the complex for the autophagic activity that appears to be essential for the benefits of MR to CLS. Indeed, this notion is consistent with our observation that *met15Δ vps34Δ* cells are exceedingly short-lived (Figure 1G). That said, another PI3K complex exists (complex II) that features Vps38 in place of the complex I-specific factor Atg14 and functions in vacuolar protein sorting rather than autophagy (Kihara et al., 2001; Obara et al., 2006). To confirm that MR-dependent CLS extension requires the autophagy-promoting activities of Vps15, Vps30, and VPS34, rather than their roles in vacuolar protein sorting, we assessed the requirement of the complex II-specific factor Vps38 for the extended CLS of cells undergoing G-MR. We found that *vps38Δ met15Δ* double mutant cells are no shorter-lived than *met15Δ* cells (Figure 1H), indicating that complex II activity is dispensable for the full extension of CLS by MR. Similarly, removal of the autophagy-related factor Iml1 from cells undergoing G-MR also fails to shorten lifespan (Figure 1I). While Iml1 is a positive regulator of autophagy that was observed to be upregulated by MR, it is part of a complex that is specifically required for non-nitrogen starvation-induced autophagy and is not necessary for autophagy under all conditions (Wu and Tu, 2011). Taken together, the above experiments are consistent with MR extending CLS, at least in part, by upregulating expression of all four members of the PI3K complex I and thereby increasing the autophagic capacity of yeast.

### Enzymatic MR Extends CLS

**FIGURE 2:**
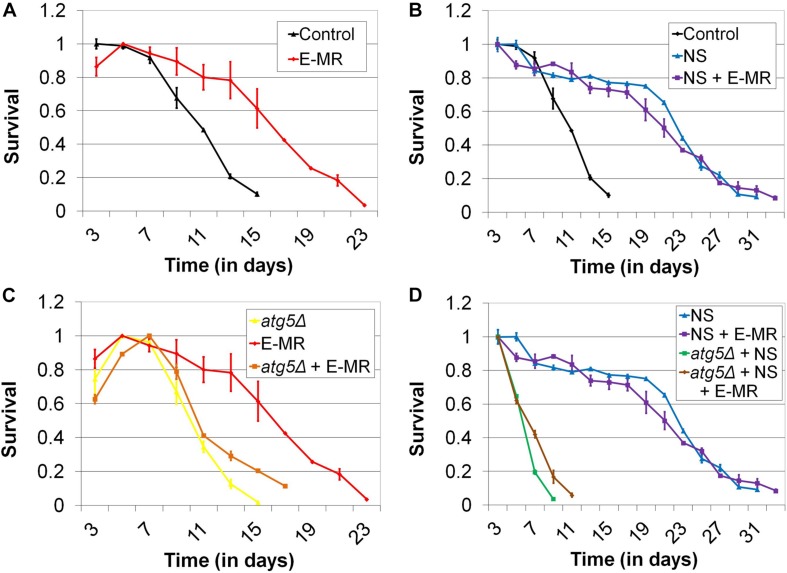
Enzymatic MR (E-MR) and nitrogen starvation both extend CLS, dependent on autophagy, and are epistatic. **(A)** Wild-type cells containing a plasmid that constitutively expresses methionine gamma lyase (i.e., E-MR) are long-lived compared with empty vector-containing control cells. **(B)** Nitrogen starvation (NS) extends CLS and is epistatic with E-MR. Extension of CLS by **(C)** E-MR and **(D)** nitrogen starvation is abrogated in autophagy-deficient (*atg5Δ*) cells. Also, autophagy-deficient cells simultaneously undergoing both E-MR and nitrogen starvation fail to receive the benefit of either intervention to CLS. Bars denote SEM.

Given that MR alters the levels of genes involved in autophagy, many of which are required for the extended lifespan of methionine-restricted cells, we sought to perform genetic epistasis studies to further explore the possibility that increased autophagic capacity underlies MR-dependent CLS extension. For this purpose, we created a novel system to produce the methionine-restricted state in wild-type cells grown in methionine-containing medium, an intervention that we term “enzymatic MR” (E-MR). Specifically, we cloned the L-methionine gamma lyase (MGL) gene from the bacterium *Clostridium sporogenes* into a construct for constitutive high-level expression in yeast. MGL is an enzyme that efficiently hydrolyzes methionine both *in vitro* and *in vivo* (Kreis and Hession, 1973a, b) and would be predicted to reduce the cellular levels of methionine in yeast containing the aforementioned plasmid. As expected, E-MR robustly extends the CLS of wild-type yeast aged in methionine-containing medium (*p* < 0.0001) (Figure 2A). Moreover, the duration of the extension is comparable to what we typically observe for either G-MR or a similar third intervention, which we previously demonstrated to extend yeast CLS and that we refer to as “D-MR” (dietary MR; i.e., growth of wild-type cells in medium lacking methionine and cysteine) (Johnson and Johnson, 2014). This demonstrates that E-MR produces the methionine-restricted state at least as efficiently as G-MR or D-MR, making it a useful tool for genetic epistasis experiments exploring the relationship between MR and autophagy activation (see below).

### MR-Dependent CLS Extension Is Epistasic With Nitrogen Starvation-Mediated CLS Extension, Both of Which Require Autophagy

To directly assess whether MR might contribute to CLS beyond effects on autophagy, we performed genetic epistasis analyses. It has been demonstrated that nitrogen starvation (i.e., incubating cells in medium lacking a key nitrogen donor) robustly activates autophagy in yeast, and that such an intervention extends CLS (Takeshige et al., 1992; Onodera and Ohsumi, 2005; Powers et al., 2006). We recapitulated this finding in our setting, observing that wild-type cells aged under nitrogen-starved (i.e., ammonium sulfate-free) conditions live approximately twice as long as cells aged in normal medium (*p* < 0.0001) (31 days vs 15 days; Figure 2B). We then aged cells undergoing E-MR under nitrogen-starved conditions and found that they are not significantly longer-lived than control cells aged under the same conditions (*p* = 0.4116) (Figure 2B). This epistatic relationship between MR and lifespan extension by nitrogen starvation provided yet more evidence that an increase in autophagic capacity underlies the benefit of MR to yeast longevity. To further these results, we deleted a gene essential for autophagy (*ATG5*) from otherwise wild-type cells and found that they fail to respond positively to E-MR (Figure 2C), nitrogen starvation (Figure 2D), or a combination of both interventions (*p* < 0.0001) (Figure 2D). Together, these results are consistent with the primary benefit of MR to chronological longevity being increased autophagic capacity.

### Activation of Autophagy by Expression of a Vps30-Derived Peptide Extends CLS

The phosphatidyl-inositol-3-kinase complex member Vps30 is the yeast homolog of mammalian Beclin1, and a study by Shoji-Kawata et al. (2013) demonstrated that administration of a peptide derived from Beclin1 (aa 267-284) to mice robustly activated autophagy *in vivo*. Mechanistically, this was achieved through the competitive binding of the peptide to GAPR-1, an inhibitor of Beclin1. Given the homology between Vps30 and Beclin1, as well as between the GAPR-1 inhibitor and the yeast factor Pry3, we hypothesized that a similar approach might be used to promote autophagy in yeast. We reasoned that should this manipulation extend CLS, it would provide more direct evidence of the causal relationship between autophagic capacity and longevity than does CLS extension by nitrogen starvation, as the latter manipulation has many other effects beyond simply promoting autophagy. We therefore generated a construct for the forced expression of a portion of Vps30 (aa 320-336) corresponding to that used by Shoji-Kawata et al. (2013) for their truncated version of Beclin1. As expected, we found that expression of Vps30^320–336^ in otherwise wild-type yeast results in a moderate extension of CLS (*p* < 0.0001) (21 days vs 17 days; Supplementary Figure S1A). Furthermore, this extension is dependent on autophagy, as expression of VPS30^320–336^ fails to extend CLS in cells lacking Atg5 (Supplementary Figure S1B). Thus, these findings provide additional evidence that promoting autophagy extends CLS and, taken with the epistasis studies of nitrogen-starved yeast, indicate that MR likely extends CLS by increasing autophagic capacity.

### Bulk Autophagy Is Not Altered by MR

**FIGURE 3:**
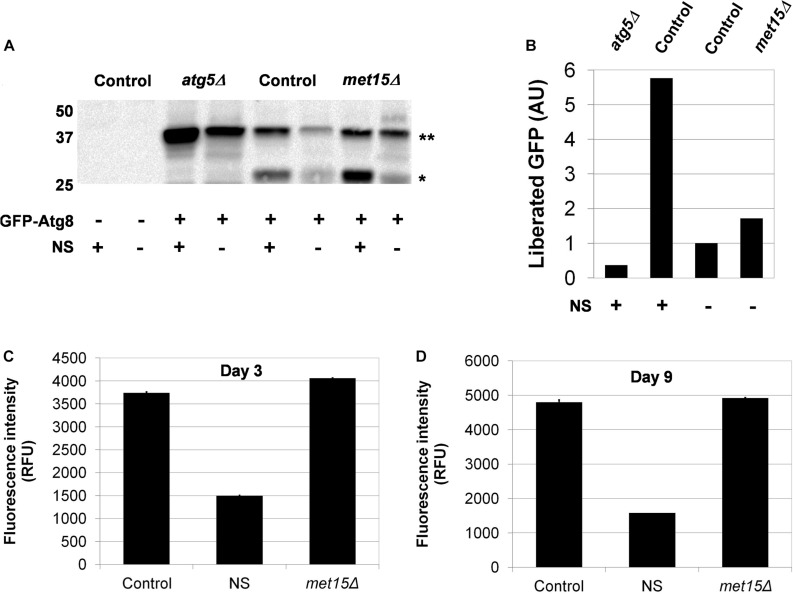
Macroautophagy does not appear to be upregulated by MR. **(A)** Direct assessment of steady-state macroautophagy in wild-type (Control), autophagy-deficient (*atg5Δ*), and methionine-restricted (*met15Δ*) cells using immunoblotting to measure the autophagic cleavage of GFP-Atg8^∗∗^ to free GFP^∗^. Samples were generated from cells containing either a GFP-Atg8-encoding plasmid or empty vector, as indicated. Nitrogen starvation (NS) was also used, as indicated, to activate autophagy. **(B)** Quantitation of the relative amounts of liberated GFP (in AUs, arbitrary units) detected by immunoblotting. Indirect assessment of steady-state macroautophagy by lipofuscin detection **(C)** early (Day 3) and **(D)** late (Day 9) during the chronological aging of wild-type and methionine-restricted cells. The fluorescence intensities of nitrogen-starved wild-type cells (NS) are included for which the activation of autophagy limits the accumulation of lipofuscin. Fluorescence intensity values are given in relative fluorescence units (RFUs). For panels **C** and **D**, bars denote SEM.

Given that genetic dependency and epistasis analyses strongly suggested an involvement of autophagy in the MR-dependent extension of CLS, we sought to determine whether the key activity was bulk autophagy (i.e., macroautophagy), one of the many selective forms of autophagy [i.e., mitophagy, pexophagy, nucleophagy, ribophagy, or the cytoplasm-to-vacuole-targeting (Cvt) pathway], or some combination thereof. To address this question, it was necessary to first assess whether steady-state macroautophagy was upregulated by MR. Toward this end, we made use of the GFP liberation assay pioneered by Daniel Klionsky’s group (Cheong and Klionsky, 2008). In this approach, autophagy-mediated processing of GFP-Atg8 results in the liberation of GFP, which in turn is measured by western blot analysis. We assessed the relative levels of free GFP in yeast undergoing G-MR (*met15Δ*) compared with control cells and found no appreciable difference in GFP liberation (Figures 3A,B). In a complementary approach, we also developed an assay to measure the accumulation of lipofuscin, autofluorescent aggregations of damaged proteins and other biomolecules that accumulate with age (Terman and Brunk, 1998; Reverter-Branchat et al., 2004). Our expectation was that an increase in macroautophagy, should it occur in methionine-restricted cells, would result in a reduced accumulation of lipofuscin. Indeed, nitrogen-starved wild-type cells (which activate autophagy and are long-lived) show consistently low levels of lipofuscin-associated fluorescence after as many as 9 days of chronological aging (Figures 3C,D). On the other hand, both wild-type and methionine-restricted cells show much greater fluorescence intensities, which increase over time. Furthermore, the levels of lipofuscin-associated fluorescence in methionine-restricted cells are not significantly different from those of wild-type controls at termination of the experiment (Day 9, *p* = 0.1931) and are actually higher, albeit marginally, at the earlier time-point (Day 3, *p* = 0.005). Thus, these cells share a similar, if not identical, degree of non-specific macroautophagic activity. Collectively, the results of the above two assays indicate that bulk autophagy is not significantly upregulated by MR.

### Mitophagy, but Not Other Forms of Selective Autophagy, Is Required for CLS Extension by MR

**FIGURE 4:**
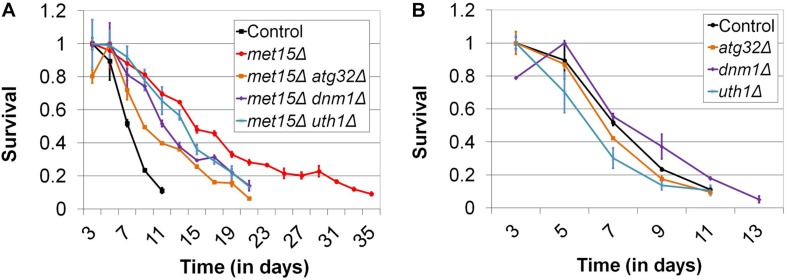
Impairment of mitophagy compromises the extended CLS of methionine-restricted yeast. **(A)** The mitophagy-related factors Atg32, Uth1, and Dnm1 are all required for the maximal extension of CLS by G-MR. **(B)** In contrast, deletion of these factors from otherwise wild-type cells does not negatively impact CLS. For both panels, Control denotes the CLS of wild-type cells and bars denote SEM.

We then considered the possibility that a selective form of autophagy (e.g., mitophagy, nucleophagy, etc.) might be active in cells undergoing MR and that this, rather than macroautophagy, might be the relevant activity underlying the extension of CLS engendered by MR. Toward this end, we identified 16 genes thought to be uniquely associated with various forms of selective autophagy, encoding factors necessary for mitophagy, pexophagy, nucleophagy, ribophagy, and the Cvt pathway, respectively. We then determined whether these genes were required for the extended CLS of methionine-restricted cells. With the exception of a subset of genes encoding factors involved in the process of mitophagy, all other genes assayed were found to be dispensable for the extended lifespan associated with MR (Supplementary Figure S2). In the case of *met15Δ* cells also lacking *ATG32* [which encodes a mitochondrial membrane protein required for the initiation of mitophagy (Kanki et al., 2009; Okamoto et al., 2009; Kondo-Okamoto et al., 2012)], maximal lifespan is impaired 43% compared with mitophagy-competent control cells undergoing G-MR (*p* < 0.0001) (21 days vs 37 days; Figure 4A and Supplementary Figure S2). Genetically methionine-restricted cells lacking Uth1, a second mitochondrial membrane protein that is indispensable for mitophagy (Kissova et al., 2004), are similarly short-lived (*p* < 0.0001) (21 days vs 37 days; Figure 4A and Supplementary Figure S2). In addition, the reduced CLS observed in these cases is not due to any putative non-specific sickness associated with these deletions, as neither *atg32Δ* nor *uth1Δ* single mutants are short-lived compared with wild-type (Figure 4B). If, as suggested, an increase in the autophagy-dependent turnover of mitochondria were to indeed underlie the extended longevity of methionine-restricted cells, it would be expected that mitochondrial fission [itself known to be required for efficient mitophagic flux (Mao and Klionsky, 2013)] would be indispensable for MR-dependent longevity. To test this, we assessed the CLS of *met15Δ* cells lacking Dnm1, a dynamin-related GTPase required for mitochondrial fission (Gammie et al., 1995; Otsuga et al., 1998; Bleazard et al., 1999), to determine how it would compare with the CLS of a control strain undergoing G-MR. Similar to the case for both the *met15Δ atg32Δ* and *met15Δ uth1Δ* double mutant strains, *met15Δ dnm1Δ* cells show a dramatically shorter CLS than *met15Δ* cells (*p* < 0.0001) (21 days vs 37 days; Figure 4A and Supplementary Figure S2). Furthermore, this reduction in CLS does not reflect any generalized sickness that might arise from deletion of *DNM1* as *dnm1Δ* single mutant cells are as long-lived as wild-type (Figure 4B). Taken together, these experiments strongly suggest that the autophagic activity underlying the extension of CLS by MR is specifically that of mitophagy, as opposed to non-specific macroautophagy or any other known form of selective autophagy.

### Mitochondrial Function Is Required for CLS Extension by MR

**FIGURE 5:**
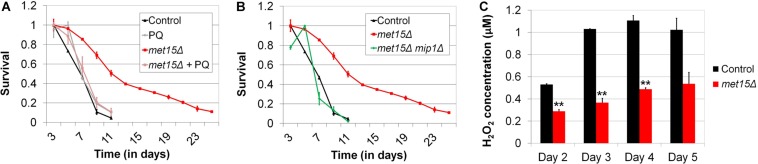
Mitochondrial function is required for MR-dependent CLS extension and likely improved by MR. **(A)** Low-level treatment (1 mM) of methionine-restricted yeast with the mitochondrial poison paraquat (*met15Δ* + PQ) reduces the CLS of such cells to that of wild-type (Control), while having no apparent effect on the longevity of wild-type cells (PQ). **(B)** Similarly, deletion of *MIP1*, which encodes a DNA polymerase required for mitochondrial function, reduces the CLS of methionine-restricted cells (*met15Δ mip1Δ*) to that of wild-type. **(C)** The levels of hydrogen peroxide present in the culture media of chronologically aging (Days 2–5) methionine-restricted cells (*met15Δ*) are approximately half of those present in the cultures of control cells. Highly statistically significant differences (*p* < 0.01) are indicated (^∗∗^) for methionine-restricted cells compared with wild-type controls. For all panels, bars denote SEM.

A critical role of mitophagy is to maintain a population of undamaged, functional mitochondria, primarily through the targeted destruction of defective mitochondria. Intriguingly, certain studies have also found that, under certain conditions, mitophagy can positively regulate *de novo* mitochondrial biogenesis (Camougrand et al., 2000; Sin et al., 2016). Given these facts, as well as the apparent involvement of mitophagy in the extension of CLS by MR, we hypothesized that mitochondrial function would be indispensable for the benefits of MR to CLS. To test this, we utilized two distinct methods to abrogate mitochondrial function, (1) treatment of cells with the mitochondrial toxin, paraquat, and (2) deletion of *MIP1*, which encodes a mitochondrial DNA polymerase that is required for maintenance of the mitochondrial genome (Genga et al., 1986; Foury, 1989). For the first method, we aged both wild-type yeast and cells undergoing G-MR in the presence and absence of paraquat. The amount of paraquat selected for these experiments (1 mM) was based on a study by Cocheme and Murphy (2008) in which this concentration was found to be the lowest that completely inhibits the growth of wild-type yeast using glycerol as a carbon source, an ability that necessitates functional mitochondria. Importantly, the authors also demonstrated that the growth of cells cultured in glucose-containing medium (i.e., the medium typically used for CLS assays) was unaffected by this level of paraquat. We observed that wild-type cells incubated in medium supplemented with paraquat are unaffected with respect to their CLS (Figure 5A). In contrast, the extended CLS of methionine-restricted cells is completely blocked by treatment with paraquat, resulting in a CLS identical to that of control cells (11 days; Figure 5A). This result is consistent with mitochondrial function being essential to the extended CLS of methionine-restricted cells, but dispensable for the normal lifespan of wild-type cells. To confirm that some aspect of mitochondrial function or metabolism is required for CLS extension by MR, we also assessed to what extent cells unable to maintain mitochondrial DNA (*mip1Δ*) display the extended longevity typically associated with G-MR. We observed that *mip1Δ met15Δ* cells are significantly shorter-lived than *met15Δ* positive control cells (11 days vs 35 days; Figure 5B), featuring a maximal lifespan identical to wild-type controls; this finding is similar to the case for methionine-restricted cells treated with paraquat, described above (Figure 5A). That said, loss of Mip1 function also results in a moderate reduction of CLS in otherwise wild-type cells (9 days vs 11 days; not shown), potentially indicating that either (a) mitochondrial function is important for the survival of wild-type yeast aged in glucose-containing medium and paraquat treatment represents a less severe mitochondrial insult than *MIP1* deletion or (b) loss of some mitochondria-independent function of Mip1 is slightly deleterious to the survival of methionine-replete cells. In either case, it remains apparent that functional mitochondria are required for the full benefit of the MR program to yeast longevity.

### The Accumulation of Reactive Oxygen Species Is Decreased by Methionine Restriction, Dependent on Mitophagy

While it is clear from the above studies that mitochondrial function is indispensable for the benefit of MR to yeast longevity, and the involvement of mitophagy suggests that methionine-restricted cells contain more functional mitochondria compared with their methionine-replete counterparts, we had yet to obtain actual evidence supporting this possibility. To test whether activated mitophagy in cells undergoing MR might result in an increase in the proportion of functional, high-quality mitochondria, we assessed the accumulation of peroxide in the culture media of both wild-type cells and those undergoing G-MR. As impairment of the electron transport chain in dysfunctional mitochondria results in the production of superoxide and hydrogen peroxide (Cocheme and Murphy, 2008; Brand, 2010, 2016), we expected that any increase in the number of functional mitochondria would result in decreased peroxide accumulation. We preferred this assay over assessing mitochondrial DNA copy number as it allows for a determination of the relative functional capabilities of the mitochondria within cells as opposed to simply determining their numbers, a measurement that would provide no information about the relative quality of these organelles. We observed that, at all time-points assessed, the levels of culture peroxide produced by cells undergoing G-MR were approximately half of those produced by control cells (Figure 5C). While the levels of peroxide detected were relatively low in both cases, suggesting the presence of high quality mitochondria present even in control cells, MR nevertheless results in a further decrease in peroxide generation. To test whether, as expected, these effects involve the mitophagic removal of defective mitochondria, we also assessed whether mitophagy-deficient (*atg32Δ*) cells are capable of lowering culture peroxide levels in response to MR (Supplementary Figure S3A). At both time-points tested, MR failed to significantly reduce the accumulation of peroxide in the cultures of mitophagy-deficient cells (Day 4, *p* = 0.0905; Day 5, *p* = 0.3137). Taken together, these data therefore suggest that mitophagic activity in methionine-restricted yeast produces an enrichment of functional mitochondria, which in turn results in more efficient respiratory metabolism.

### Altered Carbon Metabolism Underlies the Benefit of MR to CLS

**FIGURE 6:**
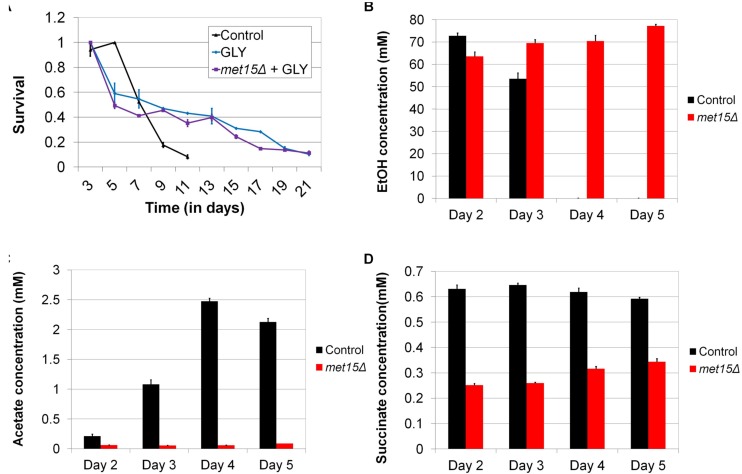
Alterations in carbon metabolism can extend CLS and are engendered by MR. **(A)** Wild-type yeast aged in medium containing glycerol in place of glucose (GLY) are long-lived, whereas methionine-restricted yeast receive no additional benefit from growth on glycerol when these interventions are combined (*met15Δ* + GLY). Determination of the varying concentrations of **(B)** ethanol, **(C)** acetate, and **(D)** succinate in chronologically-aged wild-type (Control) and methionine-restricted (*met15Δ*) yeast (Days 2–5). For panels **B–D**, all MR-dependent changes are highly statistically significant (*p* < 0.01). Bars denote SEM.

Given that mitochondria are required for aerobic respiration, as well as the extended longevity of methionine-restricted cells, we considered the possibility that MR might extend CLS by altering the metabolism of aging cells. Indeed, chronologically aging yeast have been demonstrated to utilize fermentation for energy production during their first few days of growth, after which they transition to aerobic respiration. Moreover, the timing and extent of this phenomenon, known as the “diauxic shift,” can have significant implications for chronological longevity (Fabrizio and Longo, 2003). If the benefit of MR to yeast CLS were indeed through a change in the kinetics (i.e., timing and/or efficiency) of aerobic respiration, as well as a concomitant reduction in fermentation, then one would predict (A) that cells aged in medium containing a carbon source that requires the oxidative metabolism of mitochondria for energy production might be long-lived and (B) that such an intervention would remove the relative benefit of MR. In fact, it is known from a previously published study that cells aged under such conditions (i.e., glycerol-containing medium) are long-lived compared with controls aged in glucose-containing medium (Burtner et al., 2009). To confirm this finding in our laboratory, we aged wild-type yeast in either glucose- or glycerol-containing medium, the latter of which necessitates functional mitochondria for energy production, growth, and long-term viability. As expected, cells aged in glycerol-containing medium are significantly longer-lived than those aged in glucose-containing medium (*p* < 0.0001) (21 days vs 11 days; Figure 6A). In addition, cells aged in glycerol-containing medium are all equivalently long-lived, regardless of whether or not they are methionine-restricted (*p* = 0.5016) (Figure 6A). This finding demonstrates that MR is incapable of extending the CLS of cells that rely on mitochondria for energy production, and such perfect epistasis is consistent with the model that MR extends cellular lifespan by promoting mitochondria-dependent alterations in carbon metabolism.

While the above genetic studies implicate metabolic alterations in the extension of CLS by MR, we sought to confirm such changes directly using biochemical methods. It is well documented that ethanol and acetic acid are byproducts of fermentative carbon metabolism in yeast (Dickinson and Schweizer, 2004), and so we used two colorimetric assays to measure the absolute levels of these metabolites in the cultures of control cells and cells undergoing G-MR. In this fashion, we assessed the extent to which cells utilized fermentation, rather than aerobic respiration, for energy production at various time-points. We observed that wild-type cells aged in methionine-replete medium produce a substantial quantity of ethanol (∼70 mM) early during chronological aging (Figure 6B), the levels of which gradually decrease to below detection by Day 4. This is consistent with similar analyses of ethanol production of wild-type cells undergoing chronological aging (Zampar et al., 2013). However, contrary to our expectations, the ethanol content of medium from aging cells undergoing G-MR remains relatively high (∼70 mM) for the entirety of the experiment (5 days). Given that the diauxic shift typically involves the conversion of ethanol to acetaldehyde, and then to acetate, this finding suggests that cells undergoing G-MR likely do not exhibit this conversion and should therefore accumulate relatively low levels of acetic acid. If true, such a finding would be in agreement with our previous observation that methionine-restricted cells acidify their culture medium at a reduced rate compared with methionine-replete controls (Johnson and Johnson, 2014). As expected, wild-type control cells aged in methionine-replete medium show a progressive increase in the levels of culture acetate over time (Figure 6C). Furthermore, this increase corresponds temporally with the aforementioned reduction in ethanol concentration (Figure 6B), as would be predicted given the conversion of ethanol into acetate. Strikingly, the amount of acetate produced by cells undergoing G-MR remains low at all time-points, at concentrations barely above detection (Figure 6C).

To further explore the alteration of carbon metabolism by MR, we also assessed the levels of succinate present in the cultures of aging yeast. Succinate, together with NADH, is the primary electron donors for the mitochondrial electron transport chain, and the former is produced through the combined action of the citric acid and glyoxylate cycles (Dickinson and Schweizer, 2004). Given its role as an electron donor, any change in succinate levels would be expected to be associated with altered flux through the electron transport chain. Similar to the results observed above for peroxide generation, we found that the levels of succinate present in the cultures of methionine-restricted cells are approximately half those of methionine-replete controls (Figure 6D). This reduction was observed for the entire duration of the experiment and provides yet additional evidence that yeast undergoing MR show a dramatically different program of carbon metabolism compared with that of control cells. Furthermore, it is clear that said program is marked by a drastic reduction in the accumulation of multiple toxic metabolites (i.e., acetic acid and reactive oxygen species) that limit the lifespan of yeast.

## Discussion

In previous work, we demonstrated that both G-MR and D-MR extend the CLS of yeast, identified an indispensable role for RTG signaling in this extension, and characterized transcriptional alterations that result from MR (Johnson and Johnson, 2014). While we identified several factors involved in autophagy as being differentially expressed in cells undergoing MR, suggesting a role for this process in supporting the benefits of MR, Ruckenstuhl et al. (2014) observed the presence of autophagy-dependent acidified vesicles in methionine-restricted cells. We therefore sought to further explore the involvement of autophagy in the benefits of MR to yeast, beginning by assessing whether various autophagy factors demonstrated to be upregulated by MR were indeed required for the longevity of cells subjected to this intervention. This approach implicated the activity of the PI3K complex I factors as being essential for the full benefit of MR to CLS, consistent with the role of this complex in supporting macroautophagy, as well as various forms of selective autophagy (Cebollero and Reggiori, 2009; Delorme-Axford et al., 2015). While bulk autophagy was not observed to be upregulated, genetic studies instead revealed that the increased autophagic capacity associated with elevated PI3K complex I activity in methionine-restricted cells likely supports activated mitophagy, as we found the latter to be required for the full extension of CLS by MR. That said, our finding that macroautophagy is not activated by MR does not preclude a putative role for this process (independent of supporting mitophagy) in the extension of MR-dependent CLS. Indeed, future studies might identify such a role. As of now, however, it is clear that the process of mitophagy is indispensable for the benefit of MR to CLS.

While mitophagy functions to remove aged and/or damaged mitochondria, it has also been implicated in the regulation of mitochondrial biogenesis (Camougrand et al., 2000; Sin et al., 2016). Whether by the selective elimination of dysfunctional mitochondria or the upregulation of new, undamaged mitochondria, activated mitophagy can be expected to result in an increase in the overall number of functional mitochondria, with a concomitant increase in respiratory capacity. Indeed, as evidenced by reduced peroxide accumulation, we found oxidative metabolism to be improved in methionine-restricted cells, dependent upon a functional mitophagic system. Furthermore, direct measurements of multiple metabolites in the cultures of aging yeast revealed significant alterations in carbon metabolism for cells undergoing MR, compared with methionine-replete control cells. However, unlike the case for peroxide accumulation, MR-dependent changes in the culture levels of ethanol, acetate, and succinate do not require mitophagy (Supplementary Figures S3B–D). With respect to such changes, we found MR to result in persistently high culture levels of ethanol for all time-points assessed. This is in stark contrast to control cells, which show a progressive depletion of ethanol (beginning to decrease on Day 3, and completely undetectable by Day 4), presumably as this two-carbon alcohol is converted into acetaldehyde, and subsequently into acetate, by the actions of alcohol dehydrogenase and aldehyde dehydrogenases, respectively (Bennetzen and Hall, 1982; Meaden et al., 1997; Boubekeur et al., 1999; Navarro-Avino et al., 1999; Saint-Prix et al., 2004). Formally, the observed differences in the levels of ethanol present in the cultures of methionine-restricted and methionine-replete aging yeast might be due to two differing mechanisms, (1) changes in metabolism, or (2) differential permeability/transport of these molecules across the cellular membrane. While active transporters exist for some of the metabolites assessed (e.g., acetate is actively transported by Jen1 and Ady2), ethanol has been shown to passively diffuse across the yeast plasma membrane (Guijarro and Lagunas, 1984; Paiva et al., 2004; Soares-Silva et al., 2007). Thus, the altered culture levels of ethanol resulting from MR must be due to effects on the metabolism of ethanol rather than its transport. Put another way, it is clear that MR must cause changes in central carbon metabolism that reduce the conversion of ethanol into acetaldehyde following the diauxic shift, thereby limiting the generation of acetate.

The sustained high levels of ethanol (as well as the low levels of acetate) in the culture medium of methionine-restricted cells suggests that either Adh2 activity (which is responsible for the conversion of ethanol to acetaldehyde) is absent or that the generation of ethanol from acetaldehyde (due to the action of Adh1, Adh3, Adh4, and/or Adh5) is so great as to offset the processing of this alcohol by Adh2 (Bennetzen and Hall, 1982; Young and Pilgrim, 1985; Drewke and Ciriacy, 1988; Smith et al., 2004). Interestingly, the expression of *ADH4* was found to be upregulated in methionine-restricted cells (3.5X) compared with methionine-replete controls (Johnson and Johnson, 2014). A putative decrease in flux from ethanol to acetate following the diauxic shift in methionine-restricted cells would be consistent with the measured culture levels of these metabolites. However, we consider it unlikely that an increase in alcohol dehydrogenase activity might be the sole mechanism responsible for the observed low levels of culture acetate. This is because our previous study found the gene encoding an isoform of pyruvate decarboxylase (*PDC6*) to be massively upregulated (56X) in methionine-restricted cells (Johnson and Johnson, 2014). Pdc6 is one of the three pyruvate decarboxylase enzymes that catalyze the decarboxylation of pyruvate to acetaldehyde (Hohmann, 1991). Thus, it is probable that there is significant carbon metabolic flux in methionine-restricted cells from pyruvate to acetaldehyde, a proportion of which would be expected to undergo conversion to acetate. Persistently low levels of culture acetate produced by methionine-restricted cells are likely then achieved through the increased utilization of this metabolite by the citric acid and glyoxylate cycles in the form of acetyl-CoA (which is then converted into citrate through the action of citrate synthase). Although a previous report suggested that it is actually the utilization of acetic acid that is toxic to chronologically aging yeast (Orlandi et al., 2013), our data instead indicate that biological processes that consume acetate (which thereby reduce its level) serve to protect cells against acetic acid-induced killing. For example, we find that loss of Acs2 activity from otherwise wild-type yeast dramatically sensitizes cells to short-term incubations with exogenous acetic acid (200 mM) (data not shown). Acs2 (acetyl-CoA synthetase isoform 2) generates the nuclear pool of acetyl-CoA used for histone acetylation from acetate (Guranowski et al., 1994; van den Berg et al., 1996; Takahashi et al., 2006), and decreased activity of this enzyme would be predicted to increase acetic acid levels. Therefore, Acs2 likely confers protection against acetic acid-induced killing by simply reducing acetate levels. Similarly, it is possible that, in methionine-restricted cells with elevated mitochondria-dependent oxidative metabolism, an increased demand for the electron donor succinate might contribute to the efficient depletion of the acetate pool.

**FIGURE 7:**
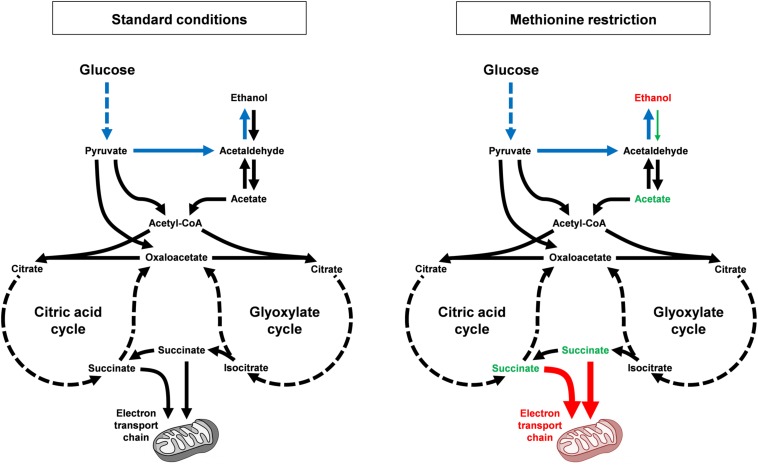
MR alters carbon metabolism and promotes longevity, dependent on mitochondrial function. Under standard growth conditions (left), budding yeast display diauxic growth, with a primary aerobic glycolysis (or fermentation) phase, wherein glucose is rapidly converted to ethanol (shown in blue; multi-step conversions are indicated by hashed lines). After all glucose is consumed, cells then convert ethanol into acetate, which is then shunted into the citric acid and glyoxylate cycles in the form of acetyl-CoA. Multiple metabolites are then produced, including succinate, which can be used as an electron donor for the mitochondrial electron transport chain. Alternative pathways exist that convert pyruvate into either acetyl-CoA or oxaloacetate without first being decarboxylated to acetaldehyde. In response to MR (right), cells show a dramatically different pattern of carbon metabolism. Ethanol levels remain persistently high (metabolites and processes that are upregulated by MR are indicated in red), apparently unable to be converted into acetate. As a result, acetate levels remains persistently low (metabolites and processes that are downregulated by MR are indicated in green). This is notable as acetic acid is the toxic intermediate that kills cells during chronological aging. Succinate levels are also low (in part due to lower steady-state levels of acetate) and the persistently low levels of both acetate and succinate are likely also due to increased consumption of these compounds by more functional mitochondria, the result of mitophagy.

Overall, our data are supportive of a model wherein MR alters carbon metabolism in at least two fashions to reduce the accumulation of toxic metabolites and improve the long-time survival of yeast (Figure 7). The first mechanism involves the reduced flux of carbon from ethanol to acetate, which is toxic to yeast in its acid form. Indeed, the persistently high levels of ethanol present in the cultures of methionine-restricted cells represent a pool of carbon that is kept unavailable for acetate production. As mentioned above, a relative reduction in the activity of a single enzyme [Adh2, the sole alcohol dehydrogenase known to be involved in conversion of ethanol to acetaldehyde (Bennetzen and Hall, 1982)] could, in theory, produce such a condition. Conversely, the inability of cells to metabolize culture ethanol might be due to more complex alterations, such as an increase in the activity of any of a multitude of other alcohol dehydrogenases (or a combination thereof). A second mechanism contributing to the reduced accumulation of acetic acid and reactive oxygen species is the mitochondria-dependent increase in efficient aerobic respiration that promotes the utilization of acetate and reduced superoxide/peroxide production. This mechanism is well-supported by our data demonstrating (A) that mitochondrial function is indispensable for MR-dependent CLS extension, (B) that dietary interventions that promote aerobic respiratory metabolism extend CLS, and (C) that such interventions show positive epistasis with MR as regards CLS. This is due, in part, to the fact that growth on glycerol (and non-fermentable carbon sources such as lactate and ethanol) promotes mitochondrial biogenesis (Ibrahim et al., 1973; Damsky, 1976; Visser et al., 1995) and that aging cells in medium containing such carbon sources extends CLS (Figure 6A) (Burtner et al., 2009). Given that such interventions are epistatic to MR with respect to positive effects on longevity, we therefore consider it highly likely that a key benefit of MR is an increase in the oxidative metabolism of mitochondria.

While carbon utilization obviously differs somewhat in mammals compared with yeast, future experiments will reveal whether alterations in the metabolism of two-carbon compounds and the activity of mitochondria are engendered in the tissues of methionine-restricted mice and/or humans, and how they might be causally related to MR-mediated improvements in organismal healthspan. Indeed, some studies have already been published that describe roles for MR in altering both glucose metabolism (Miller et al., 2005) and the production of mitochondrial reactive oxygen species (Sanz et al., 2006; Perrone et al., 2010) in rodents. While these changes are likely to benefit the healthspan of methionine-restricted animals, it will be interesting to determine precisely the extent to which they contribute to MR-dependent healthspan extension and whether it might be possible to design novel interventions (pharmacological or otherwise) that produce similar healthspan-promoting changes in the context of a normal, methionine-replete diet.

## Materials and Methods

### Yeast Strains and Plasmid Construction

All experiments were performed using haploid strains derived from the BY4741/BY4742 background (*his3Δ1, leu2Δ0, ura3Δ0*) (Brachmann et al., 1998). Specifically, strains used were from (or derived from strains obtained from) the commercially available Yeast Knockout (YKO) Collection (GE Healthcare Dharmacon; Lafayette, CO, United States). The YKO Collection comprises strains in which gene deletions of interest are marked by the KanMX drug resistance cassette (with the exception of *met15Δ0*, *his3Δ1*, *leu2Δ0*, and *ura3Δ0*. Conversely, the *iml1Δ* strain was generated by disruption of the *IML1* locus in BY4741 using an *im1Δ* mini-transposon (mTn) plasmid (GE Healthcare Dharmacon; Lafayette, CO, United States). In the resulting strain, deletion of *IML1* is marked by a 6 kb disruption cassette carrying *lacZ*, *URA3*, *tet*, and three copies of the coding region for the hemagglutinin epitope (*3X-HA*). The methionine gamma lyase and Vps30^320–336^ expression plasmids were each constructed using a combination of traditional cloning and the *Saccharomyces cerevisiae* Advanced Gateway Cloning system (Alberti et al., 2007). For the former, the *MGL* gene was PCR amplified from DNA of the bacterium *C. sporogenes* using the following primers (5′-GGGGACAAGTTTGTACAAAAAAGCAGGCTTGAAAGGG GATTAATATATGGAGA, 3′-GGGGACCACTTTGTACAAGA AAGCTGGGTAATAACAAATGTTGGTTCCTTATG). The resultant fragment was introduced into Advanced Gateway Entry Vector pDONR221 using a BP clonase recombination reaction. The *MGL* ORF was then transferred into Advanced Gateway Destination Vector pAG425GPD-ccdB (Addgene plasmid # 14154^1^; RRID:Addgene_14154) using an LR clonase recombination reaction, giving rise to a plasmid encoding methionine gamma lyase under control of the constitutively active *GPD* promoter. For the plasmid encoding the Vps30 autophagy activation peptide, the region of the *VPS30* ORF corresponding to amino acids 320-336 was PCR amplified from *S. cerevisiae* DNA using the following primers (5′-GGGGACAAGTTTGTACAAAAAAGCAGGCTTCTAGAACT AGTGGATC, 3′-GGGGACCACTTTGTACAAGAAAGCTGGG TTCTCAATCCATTCTAAGTGGCAAA). As above, the resultant fragment was introduced into Advanced Gateway Entry Vector pDONR221 using a BP clonase recombination reaction. The *VPS30^320–336^* fragment was then sub-cloned into Advanced Gateway Destination Vector pAG425GPD-EGFP-ccdB (Addgene plasmid # 14322^2^; RRID:Addgene_14322) using an LR clonase recombination reaction, giving rise to a plasmid encoding GFP-Vps30^320–336^ under control of the constitutively active *GPD* promoter. The Advanced Gateway Destination Vectors were obtained from Susan Lindquist. For GFP liberation studies (see below), we made use of the pRS416-based GFP-Atg8-expressing plasmid pGFP-ATG8(416)/GFP-AUT7(416). This plasmid was obtained from Daniel Klionsky (Addgene plasmid # 49425^3^; RRID:Addgene_49425) (Guan et al., 2001).

### Yeast Chronological Aging Assays

Chronological aging assays were performed as previously described (Johnson and Johnson, 2014), modified from the protocols of Longo et al. (1996). Briefly, cells were struck onto YPAD solid media from frozen stocks or dissection plates and allowed to grow at 30°C for 48 h before colonies were inoculated into liquid synthetic complete (SC) medium. After an additional 48 h of growth, aliquots were transferred into fresh SC medium at a concentration of ∼2 × 10^5^ cells/ml and grown at 30°C. Following 3 days of growth, after the diauxic shift, aliquots were removed at 48 h intervals and their colony forming units (CFUs) assessed on YPAD agar plates (*n* = 4 for each condition). For each culture, the point at which the remaining CFUs were found to be less than 10% of maximal was considered the end of lifespan. This cut-off was chosen so as to avoid the potential confound of the GASP (Growth Advantage in Stationary Phase) phenotype, which is marked by the cyclical growth and death of a small population of cells (Fabrizio and Longo, 2007). Aging assays were performed using SC medium formulated as follows: 0.67% yeast nitrogen base without amino acids, 2% glucose, 0.45% casamino acids, 0.01% tryptophan, 0.008% adenine sulfate, and 0.009% uridine. For experiments utilizing media lacking amino acids, the control media were formulated as follows: 0.67% yeast nitrogen base without amino acids, 2% glucose, 0.0018% adenine sulfate, 0.037% leucine, and 0.0073%, each, of the following: alanine, arginine, asparagine, aspartic acid, cysteine, glutamine, glutamic acid, glycine, histidine, inositol, isoleucine, lysine, methionine, para-aminobenzoic acid, phenylalanine, proline, serine, threonine, tryptophan, tyrosine, uridine, and valine. Amino acid-restricted media was prepared as described, but lacking the appropriate amino acids (i.e., leucine, uridine, etc.). For experiments involving nitrogen starvation-mediated autophagy activation, the medium was formulated as follows: 0.67% yeast nitrogen base without amino acids and ammonium sulfate, 2% glucose, 0.018% leucine, and 0.009%, each, of histidine, uridine, and lysine. Where required, to support the growth of genetically methionine-restricted cells (*met15Δ*), nitrogen starvation medium was supplemented with 0.009% methionine. For experiments involving the chronological aging of yeast in glycerol-containing medium, it was formulated as follows: 0.67% yeast nitrogen base without amino acids, 2% glycerol, 0.45% casamino acids, 0.01% tryptophan, 0.008% adenine sulfate, and 0.009% uridine. To assess the significance of lifespan differences between strains and/or conditions, 10% survival values (in days) were computed, and used to perform unpaired two-tailed *t*-tests. The program Prism (Graph-Pad Software; La Jolla, CA, United States) was used for all statistical analyses.

### Measurements of Autophagy by GFP-Atg8 Western Blotting and Fluorometric Analyses of Lipofuscin

To assess bulk autophagy, we made use of a method described by Daniel Klionsky’s laboratory for monitoring the autophagic degradation of a GFP-Atg8 fusion protein by immunoblotting (Cheong and Klionsky, 2008). This assay was performed essentially as described, with some minor alterations. Briefly, strains harboring either the GFP-ATG8(416)/GFP-AUT7(416) plasmid or an empty vector were chronologically aged in SC medium lacking uridine or in nitrogen starvation medium, as indicated. Cells were harvested after 7 days of aging and used to generate TCA-precipitated protein extracts. For immunoblotting, blots were incubated overnight in 1:4000 anti-GFP primary antibody (ab290; Abcam; Cambridge, United Kingdom) at 4°C, followed by an incubation for 1 h in 1:10,000 goat anti-rabbit IgG horseradish peroxidase-conjugated secondary antibody (1706515; Bio-Rad; Hercules, CA, United States) at room temperature. Blots were developed using the SuperSignal West Pico Plus Chemiluminescent Substrate (Thermo Fisher Scientific; Carlsbad, CA, United States) and visualized with a Bio-Rad ChemiDoc XRS + Molecular Imager. Image analysis and densitometry were performed using Bio-Rad Image Lab version 4.1. Normalization for variations in gel loading was performed using densitometry of representative protein bands on blots stained with Ponceau S dye (Sigma–Aldrich; St. Louis, MO, United States).

As an indirect assessment of bulk autophagy, the accumulation of fluorescent lipofuscin aggregates was measured in live yeast using a Molecular Devices SpectraMax M5 Microplate Reader (Molecular Devices LLC; San Jose, CA, United States). For each condition, approximately 100 μl aliquots of chronologically aging yeast cultures (normalized by cell density, OD^600^) were transferred to the wells of a clear, flat-bottom 96 well plate and their fluorescence intensities measured at an excitation wavelength of 350 nm and emission wavelength of 440 nm. These wavelengths were determined to be optimal for the fluorescence measurement of lipofuscin by using excitation and emission scans. All measurements were performed in duplicate and statistical analyses were performed using unpaired two- tailed *t*-tests.

### Colorimetric Determination of Culture Peroxide, Ethanol, Acetate, and Succinate Levels

Media samples used for measurement of peroxide and fermentative/respiratory metabolite levels were isolated from aging yeast cultures prepared as described in the section above concerning yeast chronological aging assays. On the indicated days, 100 μl aliquots were removed, clarified by centrifugation, and stored at −20°C. The colorimetric assays to measure peroxide (MAK311), ethanol (MAK076), acetate (MAK086), and succinate (MAK184) were obtained commercially (Sigma-Aldrich; St. Louis, MO, United States) and performed according to the manufacturer’s recommendations. In general, varying amounts of cultured media were added to multi-component enzyme reaction mixtures in clear, flat-bottom 96-well plates, incubated for the indicated durations, and their absorbances measured (peroxide—585 nm; ethanol—570 nm; acetate—450 nm; succinate—450 nm) using a Molecular Devices SpectraMax M5 microplate reader. Two biological replicates and two technical replicates were performed for each condition and statistical analyses were performed using unpaired two-tailed *t*-tests.

## Data Availability Statement

All datasets generated for this study are included in the article/Supplementary Material.

## Author Contributions

JJ and JP conceived, designed, and performed the experiments. JJ wrote this manuscript.

## Conflict of Interest

The authors declare that the research was conducted in the absence of any commercial or financial relationships that could be construed as a potential conflict of interest.

## Footnotes

1 http://n2t.net/addgene:14154

2 http://n2t.net/addgene:14322

3 http://n2t.net/addgene:49425
