# Supplementary figures and images for: Extension of Cellular Lifespan by Methionine Restriction Involves Alterations in Central Carbon Metabolism and Is Mitophagy-Dependent
Source: Front Cell Dev Biol. 2019 Nov 28;7:301. doi: 10.3389/fcell.2019.00301 (PMC6892753; doi:10.3389/fcell.2019.00301)

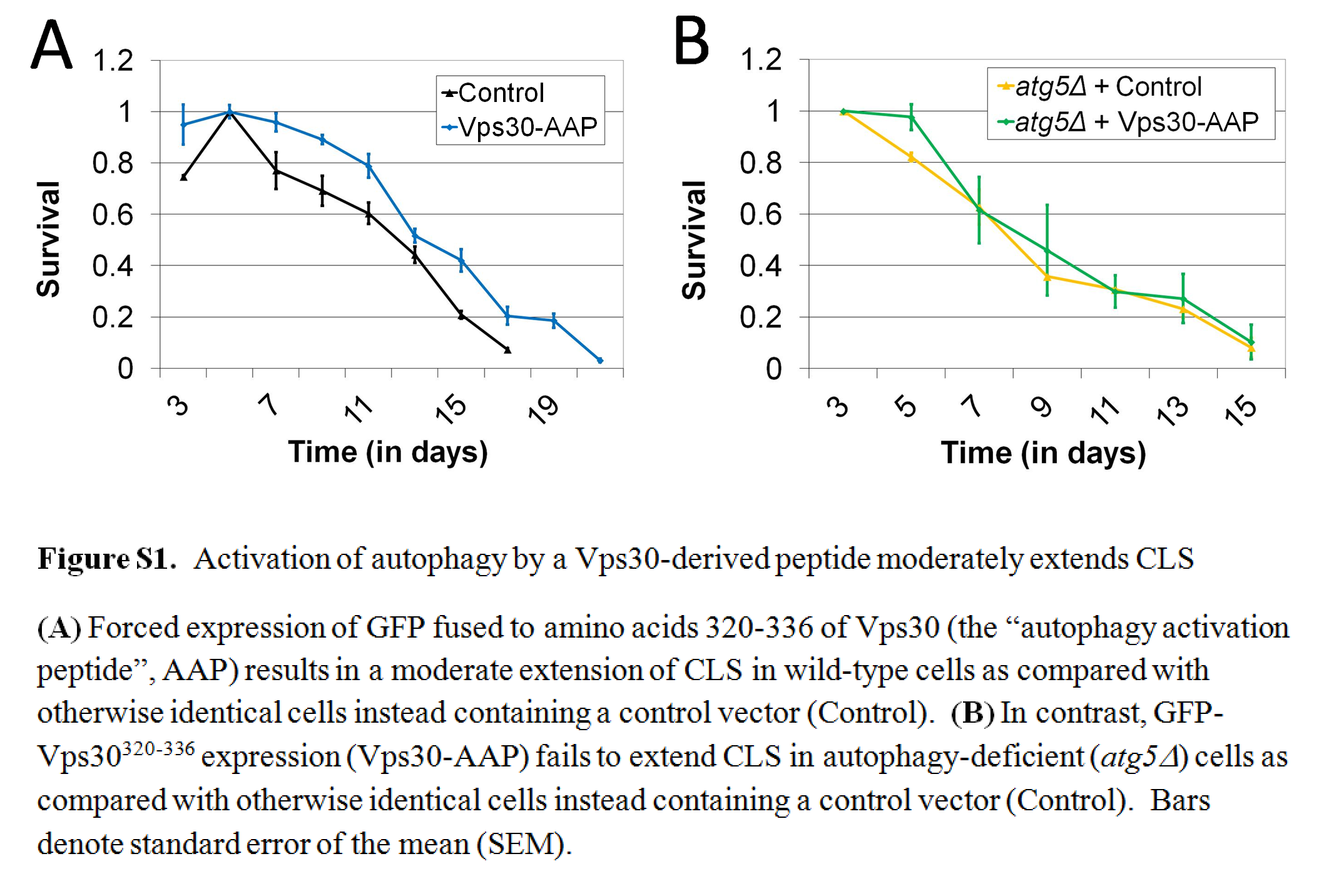

Supplement: Supplementary file 1 [file Image_1.TIF]

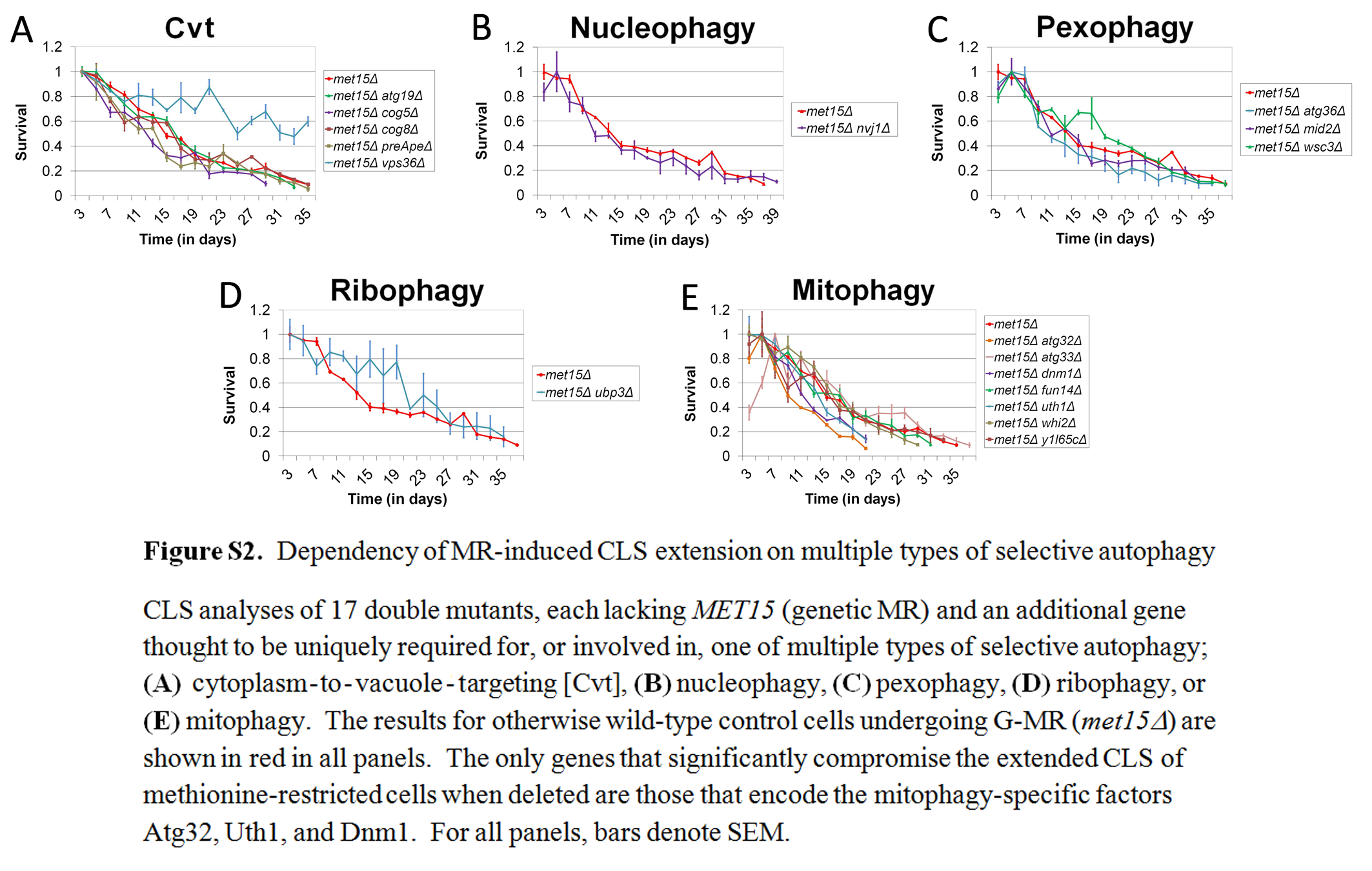

Supplement: Supplementary file 2 [file Image_2.TIF]

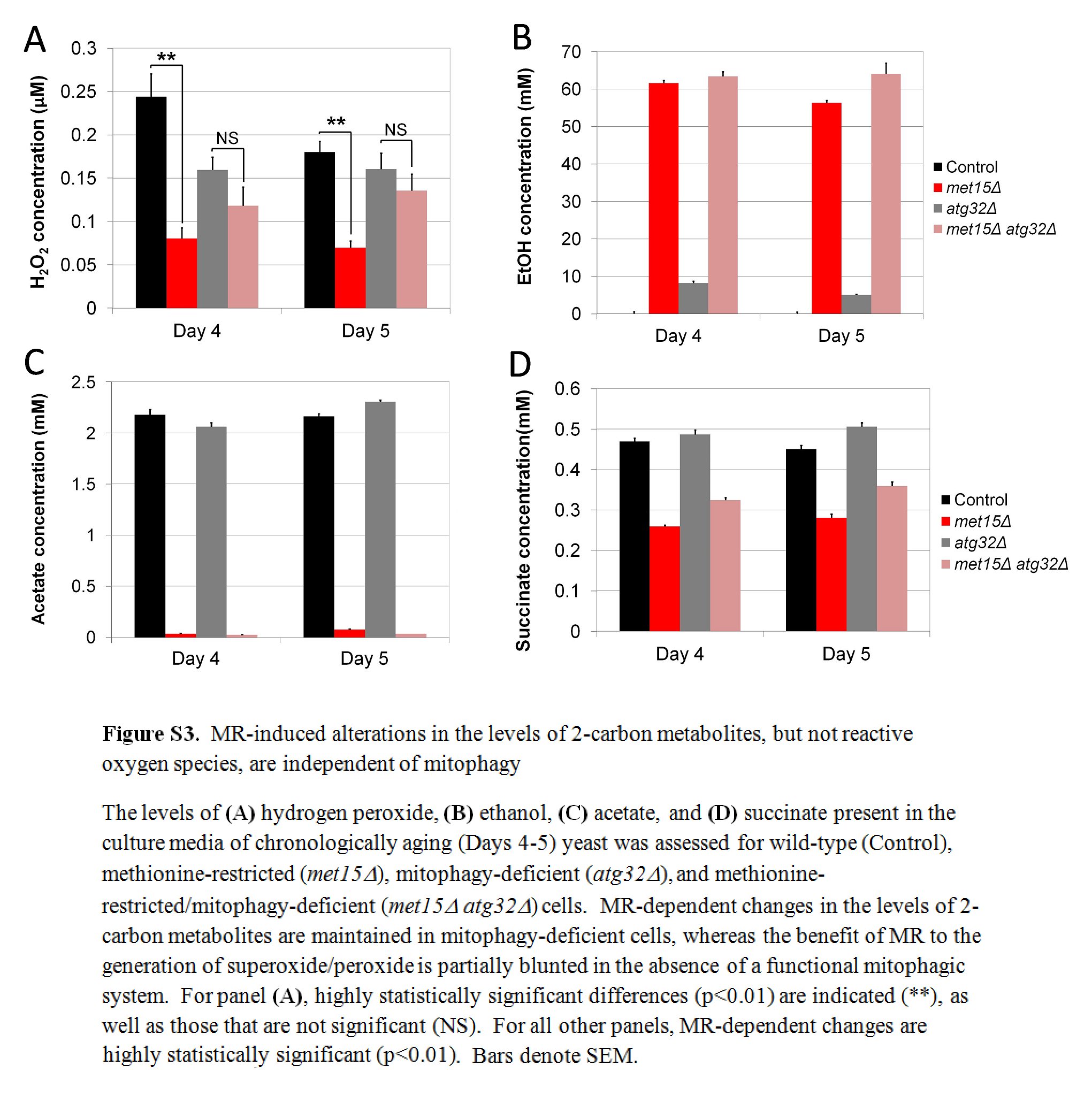

Supplement: Supplementary file 3 [file Image_3.TIF]
